# Modifiable risk factors associated with non-communicable diseases among adult outpatients in Manzini, Swaziland: a cross-sectional study

**DOI:** 10.1186/s12889-020-08816-0

**Published:** 2020-05-12

**Authors:** Mojeed Akorede Gbadamosi, Boikhutso Tlou

**Affiliations:** Discipline of Public Health Medicine, School of Nursing and Public Health, University of KwaZulu-Natal, Durban, South Africa

**Keywords:** Diabetes, Hypertension, Non-communicable diseases, Out-patients, Pre-diabetes, Risk factors, Swaziland, T2DM

## Abstract

**Background:**

Four major non-communicable diseases (NCD), including T2DM, contributed to nearly three-quarters of all deaths worldwide in 2017. Dietary and lifestyle actors associated with NCDs are potentially modifiable. Therefore, this study was conducted to determine the dietary and lifestyle factors associated with T2DM, pre-diabetes, and hypertension among adult outpatients in Manzini, Swaziland.

**Methods:**

A random sample of 385 subjects aged 18 years and above was selected. The data regarding demographics, socio-economic status, lifestyle behaviour, diet, and physical activities were collected. Additionally, participants’ anthropometric measurements and vital signs were taken. A biochemical examination was done for fasting plasma glucose, and a 2-h oral glucose tolerance test, where necessary. The Statistical Package for Social Sciences (SPSS) version 26 was used for this data analysis, and the level of statistical significance was set at *p* < 0.05.

**Results:**

A total of 385 (197 men and 188 women) subjects aged 18 years and older participated in the study. The overall prevalence of hypertension was 48.3%, while the prevalence of hypertension stage 1 and 2 were 29.4 and 19%, respectively. Smoking, SES and consumption of sweet drinks, salty processed foods, fruits, and vegetables were significantly associated with T2DM. However, in the multivariate analysis, only consumption of vegetables (*p* < 0.0001), fruits (*p* =0.014), sweet drinks (*p* = 0.042), and salty processed foods (*p* = 0.005) remained significantly associated with T2DM. Smoking (*p* = 0.002) and consumption of fruits (*p* < 0.0001), vegetables (*p* < 0.0001), and sweet drinks (*p* = 0.043) were independently associated with pre-diabetes, while the consumption of vegetables (*p* = 0.002) and salty processed foods (*p* = 0.003) were the factors independently associated with hypertension.

**Conclusions:**

The factors associated with T2DM, pre-diabetes, and hypertension are potentially modifiable. Therefore, interventions which target lifestyle changes at primary health care and population levels are warranted to address the growing burden of these chronic conditions in Swaziland.

## Background

The burden of non-communicable diseases (NCDs) in sub-Saharan Africa (SSA) is rising and is thus expected to surpass the morbidity and mortality burden due to communicable diseases (CDs) by 2030 [1]. In 2016, NCDs were the leading cause of death globally, responsible for 71% of the 57 million deaths worldwide [2]. The four leading NCDs: cardiovascular diseases (CVDs), type 2 diabetes mellitus (T2DM), chronic respiratory diseases, and cancer, jointly contributed to 78.8% of all NCD deaths worldwide [2]. Disturbingly, in 2016, 78% of all NCD deaths occurred in low- and middle-income countries (LMICs) [2].

The number of adults affected with T2DM worldwide increased from 108 million in 2008 to nearly half a billion in 2014 [3]. In 2019, about 463 million adults were affected by T2DM globally, many of the cases were reported in LMICs [4]. The highest proportional increase in the prevalence of T2DM is anticipated to occur in SSA. Numerically, about 47 million adults are estimated to have T2DM by 2045, representing a 143% increase from the 2019 figures [4]. Pre-diabetes is a significant risk factor for the development of T2DM [4–6], which is defined as an intermediate state between normal glucose homeostasis and T2DM, with a higher than normal glucose level, but below the diagnostic threshold for T2DM [6]. According to the International Diabetes Federation (IDF) [4], pre-diabetes occurs either due to impaired fasting glucose (IFG) or impaired glucose tolerance (IGT) or both. The prevalence of pre-diabetes is increasing worldwide, and it is expected that 470 million adults will develop pre-diabetes by 2030 [6]. In SSA, the prevalence of pre-diabetes will increase dramatically by 2045, rising from 45.3 million adults in 2019 to 110.2 million adults [4]. Disturbingly, Tabak et al. [6] posit that 5–10% of pre-diabetic cases per annum will progress to overt T2DM. Therefore, it is essential to identify people with pre-diabetes for timely intervention to avert its progression to T2DM.

Hypertension, also known as raised blood pressure (BP), is a significant risk factor for many diseases, such as coronary heart disease, chronic kidney disease, and strokes [7]. Globally, 25% of men and 20% of women, or one in five adults 18 years and above, had a raised BP in 2015 [8]. Although regional differences exist in the prevalence of hypertension, the highest prevalence (27%) recorded in 2016 was in Africa [8]. From 1985 to 2015, the global prevalence of raised BP has nearly doubled, with LMICs being the most affected [8]. Hypertension and T2DM are two of the metabolic risk factors, besides overweight/obesity and hyperlipidaemia, which contributes to the growing burden of NCDs in SSA [2]. Both conditions exert a substantial financial burden on individuals, families, communities, and the health system of any country [2]. Therefore, it is important to identify individuals with raised BP for lifestyle intervention, together with counselling for those individuals at risk.

According to WHO [1], NCDs are caused by four key risk factors: tobacco use, harmful use of alcohol, unhealthy diet, and physical inactivity. Alternatively, the NCD risk factors have been categorised as modifiable behavioural, non-modifiable factors (mainly age, gender, family history, ethnicity) and metabolic risk factors [2]. Timely intervention, through lifestyle modification, will avert or delay the progression of the disease in at-risk individuals or those affected by pre-diabetes, T2DM or elevated BP [1]. The benefits of lifestyle modification, such as smoking cessation, a healthy diet, moderate physical activity, and moderate alcohol use for the prevention and control of T2DM and hypertension have been well documented [6, 9–11]. Kontis et al. [11] reported, in their modelling study, that mortality due to CVDs and T2DM can be reduced by lowering the prevalence of six risk factors, which includes smoking and the harmful use of alcohol, with LMICs expected to reap most of the benefits. Studies have demonstrated the effectiveness of lifestyle modification in delaying the onset of pre-diabetes or its progression to T2DM [12, 13], as well as the control of T2DM [10], mainly through healthy diets [14].

In Swaziland, NCDs accounted for 37% of all deaths in 2016, representing a 54.2% increase from the 2014 figures [2]. According to WHO, the Swazi’s suffer more from CVD, Type-2 diabetes, cancer, and Chronic Obstructive Respiratory Diseases (COPDs) [2]. In Swaziland, hypertension was ranked first among the ten leading cases reported at outpatient departments (OPD) during 2016, while T2DM was the third leading case reported at OPDs [15]. Of note is that T2DM was the leading contributor to in-patient admissions, followed by hypertension, responsible for 15, and 11% of in-patient admissions respectively in 2016. Similarly, T2DM was the second leading cause of NCD deaths in Swaziland in 2016. Disturbingly, the mortality attributable to T2DM has been increasing steadily since 2014, rising from 47 deaths in 2014 to 109 deaths in 2016 [15].

There is a paucity of current epidemiological data on NCD risk factors, especially T2DM, pre-diabetes, and hypertension in Swaziland to be able to identify high-risk individuals for timely intervention. There is a need for epidemiological data to understand the dietary and lifestyle factors predisposing people to these chronic diseases. Therefore, this study is aimed at identifying the diet and lifestyle risk factors associated with T2DM, pre-diabetes, and hypertension among patients attending the OPD in a Manzini tertiary hospital.

## Methods

A detailed description of the examination procedures and the participants’ characteristics has been provided elsewhere [16]. Briefly, 385 patients attending the OPD at Raleigh Fitkin Memorial Hospital in Manzini, Swaziland were included in the study. Fasting blood glucose and oral glucose tolerance (OGT) testing were done using a glucometer (On Call® EZ II, ACON Laboratory, Inc., USA). Type 2 diabetes mellitus and pre-diabetes (impaired fasting glucose and impaired glucose tolerance) were defined according to WHO criteria [17]. The blood pressure measurement and definitions were made according to the American College of Cardiology/American Heart Association (ACC/AHA) guidelines [18].

Anthropometric measurements were taken and defined according to standardised criteria [19, 20]. Socio-demographic, dietary, and lifestyle variables were collected from all participants using a questionnaire. Socio-demographic variables collected included age, divided into six groups (18–27, 28–37, 38–47, 48–57, 58–67, ≥68); gender; occupation, considered as a student/unemployed, self-employed and a salaried job; and education, categorised as no formal, primary or secondary or higher education. Principal component analysis (PCA) was used to generate wealth quintiles from the asset variables. The first principal component was used to represent the wealth index, categorised into three: lower, middle, and highest SES levels. Insufficient physical activity was defined as self-reports of less than 150 min of moderate-intensity activity or less than 75 min of vigorous-intensity work, recreation, or travel per week [21].

Smoking was defined as self-reported formal or current tobacco use, including smokeless tobacco products, snuff, and pipes. Harmful alcohol use was defined as consumption of five or more standard drinks per day for men and four or more standard drinks per day for women [22]. Alcohol use included beer, wine, spirits, and a local brew (such as *buganu*). Consumption of sweet drinks was defined as self-reported consumption of sweet coffee, sweet tea, soda, and other sweet beverages per week. Rare consumption of sweet drinks was defined as the consumption of fewer than three drinks per week. Moderate sweet drink consumption was defined as a consumption of 4 to 10 drinks per week, while consumption of more than 11 sweet drinks per week was considered excessive. Salt use included the use of raw salt and salty stock cubes or powders. Consumption of salty processed foods included consumption of salty snacks, canned salty foods, salty foods prepared at fast-food restaurants. Adequate consumption of fruits and vegetables was considered as the consumption of three or more servings daily. One serving of vegetables was taken as 1 cup of raw green leafy vegetables or, half a cup of other raw or cooked vegetables (tomatoes, carrots, onions) or half a cup of vegetable juice. One serving of fruit was taken as one medium-sized apple, banana, orange, or half a cup of cooked or canned fruit or half a cup of juice from fruit (but not artificially flavoured).

### Statistical analysis

All analyses were conducted using Statistical Package for Social Sciences (SPSS) version 26 (SPSS Inc., Chicago, USA). Descriptive statistics (percentages) were used to describe the crude (unadjusted) prevalence rates for T2DM, pre-diabetes and hypertension. A Chi-square test was applied to examine the association between the independent socio-demographic, behavioural variables and pre-diabetes, T2DM, and hypertension. A binary logistic regression with a backward conditional method was performed to assess variables that could predict the development of T2DM, pre-diabetes, and hypertension. A two-sided *p*-value < 0.05 was considered statistically significant.

## Results

### General characteristics of the study population

The characteristics of the study participants, consisting of 197 (51.2%) men and 188 (48.8%) women respectively, according to diabetes status and gender, are summarised in Table 1. The prevalence of T2DM was higher among women compared to men, but pre-diabetes was more common among men than women. Both men and women with T2DM had a similar age (46 vs 45 years). Contrarily, men with pre-diabetes were younger than women with pre-diabetes (41 vs 51 years). Amongst men and women, the prevalence of abnormal glucose metabolism differed significantly with their smoking status.

**Table 1:** Characteristics of the study population according to glucose metabolism status and gender

	Men (*n* = 197)				Women (*n* = 188)			
	Normal n (%)	Pre-diabetes n (%)	T2DM ^a^ n (%)	*p*-value	Normal n (%)	Pre-diabetes n (%)	T2DM n(%)	*p*-value
n (%)	177 (46.0)	14 (3.6)	6 (1.6)		155 (40.3)	11 (2.8)	22 (5.7)	
**Mean age** (years) n (SD)^b^	35.76 (15.43)	41.36 (15.92)	46.00 (18.52)	0.138	37.53(14.51)	51.18 (18.26)	44.50 (18.48)	0.004**
**Age group** (years)				0.247				0.062
18–27	63 (92.6)	4 (5.9)	1 (1.5)		46 (86.8)	2 (3.8)	5 (9.4)	
28–37	55 (93.2)	3 (5.1)	1 (1.7)		39 (92.9)	0 (0.0)	3 (7.1)	
38–47	24 (88.9)	1 (3.7)	2 (7.4)		37 (84.1)	1 (2.3)	6 (13.6)	
48–57	11 (78.6)	3 (21.4)	0 (0.0)		15 (68.2)	4 (18.2)	3 (13.6)	
58–67	12 (75.0)	3 (18.8)	1 (6.2)		12 (70.6)	2 (11.8)	3 (17.6)	
≥ 68	12 (92.3)	0 (0.0)	1 (7.7)		6 (60.0)	2 (20.0)	2 (20.0)	
**Hypertension status**				0.334				0.248
Normal	62 (91.2)	3 (4.4)	3 (4.4)		65 (87.5)	1 (1.4)	8 (10.8)	
Elevated (Pre-hypertension)	30 (90.9)	2 (6.1)	1 (3.0)		21 (87.5)	1 (4.2)	2 (8.3)	
Stage 1	59 (93.7)	3 (4.8)	1 (1.6)		38 (76.0)	6 (12.0)	6 (12.0)	
Stage 2	26 (78.8)	6 (18.2)	1 (3.0)		31 (77.5)	3 (7.5)	6 (15.0)	
**Body mass index**				0.171				0.398
Underweight	18 (94.7)	1 (5.3)	0 (0.0)		2 (100.0)	0 (0.0)	0 (0.0)	
Normal	117 (92.1)	5 (4.0)	4 (3.2)		53 (88.3)	1 (1.7)	6 (10.0)	
Overweight	31 (79.5)	6 (15.4)	2 (5.1)		49 (77.8)	4 (6.3)	10 (15.9)	
Obese	11 (84.6)	2 (15.4)	0 (0.0)		51 (81.0)	6 (9.5)	6 (9.5)	
**Waist circumference**				0.121				0.224
Normal	156 (90.7)	10 (5.8)	6 (3.5)		43 (89.6)	1 (2.1)	4 (8.3)	
≥ 94 cm (men)/≥80 cm (women)	21 (84.0)	4 (16.0)	0 (0.0)		112 (80.0)	10 (7.1)	18 (12.9)	
**Waist-to-hip ratio**				0.069				0.832
Normal	164 (91.6)	11 (6.1)	4 (2.2)		51 (83.6)	4 (6.6)	6 (9.8)	
≥ 0.95 (men)/≥0.80 (women)	13 (72.2)	3 (16.7)	2 (11.1)		104 (81.9)	7 (5.5)	16 (12.6)	
**Education**				0.547				0.220
No formal education	9 (81.8)	2 (18.2)	0 (0.0)		10 (66.7)	2 (13.3)	3 (20.0)	
Primary	39 (86.7)	4 (8.9)	2 (4.4)		39 (76.8)	3 (5.9)	9 (17.6)	
Secondary or higher	129 (91.5)	8 (5.7)	4 (2.8)		106 (86.9)	6 (4.9)	10 (8.2)	
**Socio-economic status**				0.248				0.487
Lower	55 (87.3)	5 (7.9)	3 (4.8)		50 (78.1)	3 (4.7)	11 (17.2)	
Middle	68 (95.8)	2 (2.8)	1 (1.4)		51 (86.4)	4 (6.8)	4 (6.8)	
Higher	54 (85.7)	7 (11.1)	2 (3.2)		54 (83.1)	4 (6.2)	7 (10.8)	
**Physical activity**				0.937				0.866
No	84 (90.3)	6 (6.5)	3 (3.2)		107 (82.3)	7 (5.4)	16 (12.3)	
Yes	93 (89.4)	8 (7.7)	3 (2.9)		48 (82.8)	4 (6.9)	6 (10.3)	
**Smoking history**				0.022*				0.001*
No	91 (95.8)	3 (3.2)	1 (1.1)		89 (89.0)	0 (0.0)	11 (11.0)	
Yes	86 (84.3)	11 (10.8)	5 (4.9)		66 (75.0)	11 (12.5)	11 (12.5)	
**Alcohol use**				0.740				0.570
No	68 (91.6)	4 (5.4)	2 (2.7)		60 (85.7)	4 (5.7)	6 (8.6)	
Yes	109 (88.6)	10 (8.1)	4 (3.3)		95 (80.5)	7 (5.9)	16 (13.6)	

^a^ T2DM, Type 2 diabetes mellitus.

^b^ SD, Standard deviation.

**p < 0.05 according to univariate ANOVA

* *p* < 0.05 according to chi square Pearson statistic or Likelihood ratio

*n* Number

Table 2 shows the characteristics of the study participants according to blood pressure (BP) status and gender. The overall prevalence of hypertension was 48.3% (see Supplementary file 1); the prevalence of stage 1 and stage 2 was 29.4 and 19.0%, respectively, with 14.8% of all participants identified as having elevated BP (prehypertension). The prevalence of hypertension (24.9%) was higher among men than women (19.2%), with hypertensive men being significantly older than hypertensive women (45 vs 41 years). More men had an elevated BP than women (8.6% vs 6.2%), but women with an elevated BP had a similar age compared with male counterparts (34 vs 33 years). In both genders, raised BP varied significantly by age, BMI, and waist circumference.

**Table 2:** Gender distribution of study participants and prevalence of raised blood pressure

	Men (*n* = 197)				Women (*n* = 188)			
	Normal n (%)	Elevated BP n (%)	HTN n (%)	*p*-value	Normal n (%)	Elevated BP n (%)	HTN n (%)	*p*-value
n (%)	68 (17.7)	33 (8.6)	96 (24.9)		74 (19.2)	24 (6.2)	74 (19.2)	
**Mean age** (years) n (SD)^b^	31.79 (13.08)	32.70 (15.83)	41.07 (16.03)	< 0.0001**	34.12 (12.54)	34.42 (13.14)	44.53 (16.79)	< 0.0001**
**Age group** (years)				0.006*				0.005*
18–27	32 (47.1)	15 (22.1)	21 (30.9)		27 (50.9)	10 (18.9)	16 (30.2)	
28–37	21 (35.6)	9 (15.3)	29 (49.2)		22 (52.4)	5 (11.9)	15 (35.7)	
38–47	7 (25.9)	4 (14.8)	16 (59.3)		14 (31.8)	5 (11.4)	25 (56.8)	
48–57	3 (21.4)	2 (14.3)	9 (64.3)		7 (31.8)	2 (9.1)	13 (59.1)	
58–67	4 (25.0)	0 (0.0)	12 (75.0)		3 (17.6)	2 (11.8)	12 (70.6)	
≥ 68	1 (7.7)	3 (23.1)	9 (61.2)		1 (10.0)	0 (0.0)	9 (90.0)	
**Body mass index**				0.010*				0.008*
Underweight	11 (57.9)	1 (5.3)	7 (36.8)		1 (50.0)	0 (0.0)	1 (50.0)	
Normal	46 (36.5)	26 (20.6)	54 (42.9)		35 (58.3)	7 (11.7)	18 (30.0)	
Overweight	10 (25.6)	4 (10.3)	25 (64.1)		22 (34.9)	6 (9.5)	35 (55.6)	
Obese	1 (7.7)	2 (15.4)	10 (76.9)		16 (25.4)	11 (17.5)	36 (57.1)	
**Waist circumference**				0.016*				0.005*
Normal	65 (37.8)	29 (16.9)	78 (45.3)		28 (58.3)	6 (12.5)	14 (29.2)	
≥ 94 cm (men)/≥80 cm (women)	3 (12.0)	4 (16.0)	18 (72.0)		46 (32.9)	18 (12.9)	76 (54.3)	
**Waist-to-hip ratio**				0.738				0.810
Normal	63 (35.2)	29 (16.2)	29 (16.2)		22 (36.1)	8 (13.1)	31 (50.8)	
≥ 0.95 (men)/≥0.80 (women)	5 (27.8)	5 (27.8)	4 (22.2)		52 (40.9)	16 (12.6)	59 (46.5)	
**Education**				0.100				0.112
No formal education	2 (18.2)	3 (27.3)	6 (54.5)		4 (26.7)	1 (6.7)	10 (66.7)	
Primary	21 (46.7)	3 (6.7)	21 (46.7)		17 (33.3)	4 (7.8)	30 (58.8)	
Secondary or higher	45 (31.9)	27 (19.1)	69 (48.9)		53 (43.4)	19 (15.6)	50 (41.0)	
**Socio-economic status**				0.388				0.289
Lower	25 (39.7)	12 (19.0)	26 (41.3)		25 (39.1)	4 (6.2)	35 (54.7)	
Middle	23 (32.4)	14 (19.7)	34 (47.9)		25 (42.4)	8 (13.6)	26 (44.1)	
Higher	20 (31.7)	7 (11.1)	36 (57.1)		24 (36.9)	12 (18.5)	29 (44.6)	
**Physical Activity**				0.740				0.582
No	30 (32.3)	15 (16.1)	48 (51.6)		54 (41.5)	15 (11.5)	61 (46.9)	
Yes	38 (36.5)	18 (17.3)	48 (46.2)		20 (34.5)	9 (15.5)	61 (46.9)	
**Smoking history**				0.996				0.542
No	33 (34.7)	16 (16.8)	46 (48.4)		40 (40.0)	15 (15.0)	45 (45.0)	
Yes	35 (34.3)	17 (16.7)	50 (49.0)		34 (38.6)	9 (10.2)	45 (51.1)	
**Alcohol use**				0.240				0.395
No	31 (41.9)	11 (14.9)	32 (43.2)		31 (44.3)	10 (14.3)	29 (41.4)	
Yes	37 (30.1)	22 (17.9)	64 (52.0)		43 (36.4)	14 (11.9)	61 (51.7)	

*HTN* Hypertension; *n* Number; *SD* Standard deviation; *p < 0.05 according to Pearson or Likelihood ration chi square; **p < 0.05 according to Univariate ANOVA.

A gender differential was observed in the lifestyle, dietary habits, physical activity, and anthropometric variables (See Supplementary File 1). Significantly more women were physically less active than men (69.1% vs 47.2%, *p* < 0.0001). A higher proportion of women consumed salty processed foods (35.6% vs 32.0%), more regularly (22.4% vs 19.3%) compared to men. A similar pattern was observed in the distribution of excessive salt use (33.1% vs 24.4%). Also, fewer women than men consumed at least three servings of vegetables per day (74.5% vs 78.5%). More men were reportedly current tobacco users than women (18.8% vs 17.6%), while a higher proportion of men consumed more sweet drinks than women (30% vs 26.1%).

### Risk factors for pre-diabetes and T2DM

In the binary logistic regression models (with a backward conditional elimination method), the absence of the condition was used as a reference group. The results of the analyses are presented in Table 3. The bivariate analysis comparing the effect of each of the risk factors on the risk of developing pre-diabetes revealed that secondary or higher education, previous or current smoking status, consumption of salty processed foods, consumption of sweet drinks, and vegetable consumption were risk factors significantly associated with pre-diabetes. Possession of secondary or higher education and consumption of fruits and vegetables reduced the risk of pre-diabetes, whereas smoking and consumption of salty processed foods and sweet drinks increased the risk of pre-diabetes.

**Table 3:** Crude and adjusted odds ratios for risk factors associated with pre-diabetes and T2DM

	Pre-diabetes				T2DM			
	COR	95% CI	AOR	95% CI	COR	95% CI	AOR	95% CI
**Education**								
No formal education	1				1			
Primary	0.426	0.11–1.61			0.893	0.23–3.52		
Secondary or higher	0.283*	0.09–0.95			0.377	0.10–1.43		
**SES**								
Lower	1				1			
Middle	0.662	0.22–1.97			0.315*	0.11–0.90		
Higher	1.337	0.52–3.46			0.625	0.26–1.51		
**Physical activity**^a^								
No	1				1			
Yes	1.250	0.55–2.82			0.642	0.28–1.46		
**Smoking history**								
No	1		1		1			
Yes	8.684***	2.55–29.58	8.895*	2.27–34.82	1.579	0.73–3.44		
**Alcohol use**								
No	1				1			
Yes	1.333	0.56–3.18			1.569	0.67–3.67		
**Sweet drink consumption**								
No	1		1		1		1	
Yes	2.516	0.98–6.46	3.479*	1.08–11.65	1.986	0.85–4.64	3.185*	1.04–4.75
**Salty processed food**								
No	1				1		1	
Yes	4.048*	1.48–11.04			8.434**	2.50–28.48	7.01*	1.77–5.72
**Fruits consumption**								
No	1		1		1		1	
Yes	0.206***	0.09–0.48	0.261*	0.09–0.76	0.122***	0.05–0.30	0.120***	0.04–0.38
**Vegetables consumption**								
No	1		1		1		1	
Yes	0.048***	0.02–0.14	0.048***	0.02–0.15	0.032***	0.01–0.10	0.047***	0.01–0.16

*AOR* Adjusted odds ratio; 95% *CI* 95% Confidence interval; *COR* Crude odds ratio; *SES* Socio-economic status.

^a^ Participants meet the WHO recommended physical activity per week.

*P < 0.05, **P < 0.001, ***P < 0.0001

Participants with a secondary or higher education had a 28.3% reduction in the risk of developing pre-diabetes compared to those without formal education (crude odds ratio (COR) 0.283; 95% CI 0.09, 0.95, *p* = 0.04). This association was observed in the multivariate analysis but was attenuated by the consumption of sweet drinks, smoking, fruits, and vegetable consumption. Similar results were obtained for the association between consumption of salty processed foods and pre-diabetes in the multivariate analysis. No significant association was observed between consumption of salty processed foods and the risk of pre-diabetes. However, participants who consumed salty processed foods moderately or regularly were at a 48% increased risk of pre-diabetes compared to their counterparts who rarely consumed processed foods.

The significant association between the consumption of vegetables and pre-diabetes status remained after adjusting for smoking, consumption of salty processed foods, and consumption of fruits. Interestingly, the value of adjusted odds ratio (AOR) remained the same as the COR value; participants who consumed at least three or more servings of vegetables per day were at a 5% reduced risk of pre-diabetes compared to participants who did not (AOR 0.048; 95% CI 0.02, 0.15; *p* < 0.0001). Also, participants who consumed at least three servings of fruits daily had a 26% reduction in the risk of developing pre-diabetes compared to those who did not. The AOR improved by 5.5% from the crude odds ratio value when sweet drinks, vegetables and smoking were controlled for in the multivariate analysis. Previous and current smoking status is associated with a 790% increased risk of pre-diabetes, with a slight change in the odds ratio (AOR 8.895; 95% CI 2.27, 34.82; *p* = 0.002). Consumption of sweet drinks was not significantly associated with pre-diabetes in the bivariate analysis. Interestingly, it had a significant association with pre-diabetes in the multivariate model after adjusting for smoking, fruits, and vegetable consumption. Consumption of sweet drinks was significantly associated with a 248% increased risk of developing pre-diabetes (AOR 3.479; 95% CI 1.04, 11.65; *p* = 0.043).

In the bivariate analysis, only socio-economic status (SES) was significantly associated with T2DM risk, with participants in the middle SES stratum having a 32% reduction in the risk of developing T2DM compared to those in the lowest SES stratum. However, this association was no longer significant after adjustment was made for sweet drinks, salty processed food, fruit and vegetable consumption in the multivariate analysis. Consumption of fruits and vegetables was protective of T2DM risk, whereas consumption of sweet drinks and salty processed foods was associated with an increased risk of developing T2DM. Participants who consumed at least three servings each of fruits and vegetables daily had a reduced the risk, 12 and 5% respectively, of developing T2DM compared to those who did not. Contrarily, participants who consumed salty processed foods moderately and regularly were at a 600% increased risk of developing T2DM, compared to those who consumed these foods rarely. Similarly, participants who consumed sweet drinks moderately and excessively were at 219% increased risk of T2DM compared to their counterparts who did not.

### Risk factors for elevated BP and hypertension

Table 4 shows the risk factors associated with an elevated BP and hypertension in both bivariate and multivariate analyses. The consumption of vegetables and salty processed foods were individually significantly associated with hypertension in the multivariate analysis. Participants who consumed at least three servings of vegetables daily had a 42% reduced risk of developing hypertension, compared to those who did not. On the other hand, the consumption of salty processed foods was associated with a 110% increased risk of hypertension. Moderate and regular alcohol use was associated with a 59% increased risk of developing hypertension compared to non-alcohol use in the bivariate analysis, but this association was not significant in the multivariate analysis.

**Table 4:** Crude and adjusted odds ratio of factors associated with elevated BP and hypertension

	Prehypertension				Hypertension			
	COR	95% CI	AOR	95% CI	COR	95% CI	AOR	95% CI
**Education**								
No formal education	1		1		1			
Primary	0.276	0.06–1.24	0.284	0.06–1.27	0.503	0.18–1.41		
Secondary or higher	0.704	0.19–2.62	0.711	0.19–2.64	0.455	0.17–1.21		
**SES**								
Lower	1				1			
Middle	1.432	0.67–3.05			1.025	0.60–1.75		
Higher	1.349	0.62–2.94			1.211	0.71–2.07		
**Physical activity**^a^								
No	1				1			
Yes	1.303	0.70–2.42			1.023	0.65–1.60		
**Smoking history**								
No	1				1			
Yes	0.887	0.48–1.64			1.104	0.71–1.71		
**Alcohol use**								
No	1				1			
Yes	1.329	0.71–2.50			1.588*	1.01–2.49		
**Salt use**								
No	1				1			
Yes	0.993	0.54–1.84			1.624*	1.04–2.54		
**Salty processed food**								
No	1				1		1	
Yes	0.672	0.36–1.26			2.248***	1.44–3.52	2.100*	1.32–3.34
**Fruits consumption**								
No	1				1			
Yes	1.040	0.53–2.06			0.688	0.43–1.10		
**Vegetables consumption**								
No	1				1		1	
Yes	0.941	0.40–2.20			0.368***	0.21–0.64	0.422*	0.24–0.75

*AOR* Adjusted odds ratio; 95% *CI*, 95% Confidence interval; *COR* Crude odds ratio; *SES* Socio-economic status.

^a^ Participants meet the WHO recommended physical activity per week.

*P < 0.05, **P < 0.001, ***P < 0.0001

Backward conditional.

Surprisingly, none of the risk factors were significantly associated with an elevated BP in both bivariate and multivariate analyses. In the multivariate analysis, however, primary and secondary or higher education were individually associated with lower odds of elevated BP. Participants with primary and secondary or higher educational qualifications had a 28 and 71% lower risk, respectively, of elevated BP compared with participants without a formal education.

## Discussion

The present study assessed the modifiable risk factors associated with pre-diabetes, T2DM, and hypertension among adult outpatients in Manzini, Swaziland. Consumption of vegetables was independently associated with a lower risk of T2DM, pre-diabetes, and hypertension. The consumption of sweet drinks was an independent and significant determinant of T2DM and pre-diabetes, while the consumption of salty processed foods was significantly associated with T2DM and hypertension risk. Smoking was a significant determinant of pre-diabetes. In the univariate models, socio-economic status, education, and alcohol use were significantly associated with T2DM, pre-diabetes, and hypertension. Significant gender differences were observed in the prevalence of T2DM, pre-diabetes, and hypertension.

The protective impact of vegetable consumption on the risk of T2DM, pre-diabetes, and hypertension observed in the present study is expected, and is consistent with reports from Sub-Saharan Africa and elsewhere [23–25]. Studies have shown that the adequate consumption of fruit and vegetables reduces T2DM [23, 24], hypertension and cardiovascular incidences [26] as they are high in fibre and other micronutrients, but low in glycaemic load and energy density [23, 24]. Surprisingly, the inverse relationship observed between the consumption of fruits, and the risk of hypertension did not reach the level of statistical significance — the reason for this warrants further investigation.

The consumption of sweet drinks, which included sweet coffee, sweet tea, soda, and other sweetened beverages, was independently significantly associated with T2DM and pre-diabetes in the present study, consistent with previous reports elsewhere [27, 28]. In a prospective study of more than 50,000 women, Schulze et al. [29] found an 83% increased risk of developing T2DM for those who consumed ≥1 sweet drink per day compared with those who consumed < 1 sweet drink per month. Unlike the present study and that of Schulze et al. [29], a study by Malik et al. [30] did not observe a significant association between the consumption of sweet drinks and the risk of T2DM. It is suspected that the age differential may have played a role since the majority of the consumers in Malik et al.’s study [30] were young adults. More importantly, the fructose component of sugar in sweet drinks is considered a singularly harmful macronutrient and has been suggested to lead to obesity, hyperlipidemia and insulin resistance, key risk factors for T2DM and CVDs [31].

In the present study the consumption of salty processed foods was a significant determinant of T2DM, pre-diabetes, and hypertension. Like the present study, evidence elsewhere has shown a positive relationship between the consumption of salty processed food and T2DM [32] and hypertension [33]. In the present study, individuals who consumed salty processed foods moderately or regularly were seven times more likely to develop T2DM or pre-diabetes and twice as likely to develop hypertension, compared to those consumers who did so rarely. A possible explanation for this is that the consumption of processed food, high in non-water-soluble fats, contributed to the weight gain observed among the participants (reflected in the obesity indices). It is well known that obesity is the most important risk factor for T2DM, pre-diabetes and hypertension [2]. The influence of the consumption of salty processed foods on the NCD risk is a scarcely investigated subject in SSA, therefore more studies are needed to understand the mechanisms linking the consumption of salty processed foods to cardio metabolic diseases.

In the present study smoking was an independent determinant of pre-diabetes, consistent with previous reports from SSA and elsewhere [34, 35]. Individuals in the present study with a previous or current smoking history were eight times more likely to develop pre-diabetes, compared to non-smokers. Studies have reported a positive association between smoking and insulin resistance [36, 37]. The nicotine present in cigarettes induces a sympathetic discharge and raises the levels of antagonistic hormones, which reduces the insulin action. Thus, smoking elevates glucose levels in the body through reduced insulin production and reduced insulin action [37]. Surprisingly the association between smoking and a T2DM risk was not statistically significant in the multivariate analysis. Nevertheless, a smoking cessation intervention is warranted in this population to prevent the progression of T2DM in patients with elevated blood glucose levels.

Household wealth and education were the socio-economic variables independently associated with T2DM and pre-diabetes, respectively. Respondents from a lower SES household were significantly at risk of T2DM, whereas a mid-level SES was significantly associated with a reduced T2DM risk, consistent with findings elsewhere [38, 39]. However, this finding is contrary to the positive association found among outpatients in Ghana [25]. Recent epidemiological studies in SSA have observed an increased risk of T2DM among individuals from high SES households [40–42]. Prospective studies are needed in SSA to understand the influence of SES on the risk of T2DM, pre-diabetes and hypertension are SSA. In a Kenyan study by Mohammed et al., a primary education was inversely associated with pre-diabetes [43]. In the present study, participants with a secondary or higher education were at a 31.5% reduced risk of developing pre-diabetes. Mohammed et al. found a lower risk of developing pre-diabetes among participants with a primary education in Kenya [43]. Studies conducted in Europe have shown the beneficial influence of a formal education on the risk of diabetes [44, 45]. This finding shows the importance of education in reversing pre-diabetes to prevent its progression into full T2DM.

Moderate or excessive consumption of alcohol was significantly associated with the risk of hypertension in the present study. The Stepwise approach to surveillance (STEPS) survey [46] in Swaziland identified alcohol as a significant risk factor for NCDs, including hypertension. Like the present study, Peer et al. [47] found an increased risk of hypertension among black Southern Africans with excessive alcohol use. Excessive consumption of alcohol is a known risk factor for hypertension, while the moderate use of alcohol has a protective effect on hypertension [48]. There is a need to address the harmful use of alcohol in this setting.

Physical inactivity is an independent predictor of T2DM, pre-diabetes and hypertension [49]. Surprisingly, physical inactivity was not significantly associated with T2DM, pre-diabetes, and raised BP in this study. This finding is consistent with findings from Nigeria [50] but differs from a report which found physical inactivity is associated with an increased risk of HTN [51]. Nevertheless, the promotion of recommendations by the WHO on physical activity for health should be sustained.

The prevalence rate (6.5%) estimated for pre-diabetes (IFG/IGT) in the present study is lower than 9.8%, as previously reported in Swaziland in 2015 [46], possibly due to the higher cut-off used in the present study. Tabak et al. [6] indicated that 5–10% of individuals with pre-diabetes might progress to the T2DM stage, and are at risk of macrovascular complications, including CVD [52]. Nevertheless, evidence suggests that the beneficial impact of lifestyle modification allows many individuals with pre-diabetes to either prevent or delay the onset of T2DM [6, 13–15]. Therefore, individuals with pre-diabetes should be targeted for lifestyle intervention strategies to prevent escalating the T2DM burden in Swaziland.

The present study affirms the sex differences reported previously in SSA concerning the distribution of pre-diabetes, T2DM, and hypertension [42, 50]. The differences found in the sex distribution of chronic diseases in the present study may have been due to the gender differences observed in the behavioural variables between men and women. More women tended to be physically inactive, overweight and obese compared to their male counterparts. Therefore, gender must be considered when devising a lifestyle intervention to prevent and control these diseases. The high burden of T2DM, pre-diabetes, elevated BP and hypertension observed in the present study imply a potential rise in complications due to these chronic diseases, since many of the participants were unaware of their condition before participating in the present study. It is known that individuals with hyperglycaemia and hypertension are at higher risk of cardiovascular diseases such as stroke, IHD and chronic kidney disease [52]. Presently, cardiovascular diseases are among the leading causes of death in Swaziland [2, 15]. Therefore, the burden of CVDs could escalate unless the rising burden of its risk factors, like T2DM, pre-diabetes, and hypertension are controlled. Robust public health lifestyle interventions targeting individuals at risk of pre-diabetes, T2DM and hypertension are warranted. Such interventions should address the harmful use of alcohol and excessive consumption of sweetened beverages and of salty processed foods, including excessive raw salt use. Moreover, public health education and health promotion are needed to emphasise the beneficial influence of fruit and vegetable consumption, moderate physical activities, and the cessation of smoking.

### Study strengths and limitations

The cross-sectional nature of this study was a major limitation. Causal inference between exposure and disease outcomes is precluded. Population-based prospective studies on the influence of modifiable risk factors on the burden of NCDs is therefore warranted in Swaziland. Similarly, the use of the self-reported history on the use of tobacco and alcohol, dietary habits, and physical activity may have subjected the findings to recall bias. This potential bias may partly explain the lack of significant associations observed between physical activity and the NCDs investigated.

Despite the above limitations, this study has highlighted the rising burden of diabetes and hypertension in this public health facility in Swaziland. These findings may assist the hospital management to provide better care for the patients. It may also serve as a useful tool for the Ministry of Health in the planning of intervention programmes to control these NCDs in Swaziland. This study examined the influence of lifestyle, diets, and socio-economic status on the risk of T2DM, pre-diabetes, and hypertension. To the researcher’s knowledge, no study has examined the impact of modifiable risk factors on chronic diseases in this health facility or in Swaziland.

This study highlights the need for a multi-faceted and multi-sectoral approach to lifestyle modifications to arrest the growing burden of chronic diseases in Swaziland. Public health policymakers, programme managers, and health care practitioners in Swaziland need to promote the benefits of proper nutrition and healthy lifestyles. Health policymakers need to develop strategies to promote a healthy lifestyle among the general population. The government needs to introduce policies to regulate the negative influence of transnational food companies and limit the harmful use of alcohol. Also, the government needs to demonstrate its commitment to tobacco control, notably banning tobacco advertisements.

## Conclusions and recommendations

In conclusion, the consumption of fruits, vegetables, sweet drinks, and salty processed foods were the independent risk factors associated with T2DM and pre-diabetes (except for processed foods). Consumption of vegetables was protective against the risk of hypertension, whereas smoking was associated with increased odds of hypertension. The prevalence of hypertension was higher than in previous reports, and the high prevalence of elevated BP and pre-diabetes require urgent lifestyle intervention. Therefore, health planners and policymakers in Swaziland and SSA must implement lifestyle modification interventions, while the activities of the transnational companies and tobacco advertisers should be adequately regulated.

Further longitudinal studies are urgently needed to examine the impact of modifiable risk factors on the risk of NCDs, to address the growing burden of NCDs in Swaziland.

## Supplementary information

**Additional file1: Supplementary file 1.** Gender distribution of hypertension, lifestyle and anthropometric variables

## Data Availability

All data generated or analysed during this study are included in this published article [and its supplementary information file].

## Abbreviations

BP: Blood pressure
NCD: Non-communicable diseases
SSA: Sub-Saharan Africa
T2DM: Type 2 diabetes mellitus
SES: Socio-economic status
