# Supplementary material for: Modifiable risk factors associated with non-communicable diseases among adult outpatients in Manzini, Swaziland: a cross-sectional study
Source: BMC Public Health. 2020 May 12;20:665. doi: 10.1186/s12889-020-08816-0 (PMC7216325; doi:10.1186/s12889-020-08816-0)
Supplement: Supplementary file 1 — Additional file1: Supplementary file 1. Gender distribution of hypertension, lifestyle and anthropometric variables [file 12889_2020_8816_MOESM1_ESM.docx]

**Supplementary file 1. Gender distribution of hypertension, lifestyle and anthropometric variables**

**Table A.** **Gender distribution of hypertension**

| **Variable** | **Total** | **Men** | **Women** | ***p*-value** |
| --- | --- | --- | --- | --- |
| **Hypertension** |  |  |  |  |
| Normal | 142 (36.9) | 68 (34.5) | 74 (39.4) | 0.304 |
| Elevated BP | 57 (14.8) | 33 (16.8) | 24 (12.8) |  |
| Stage 1 | 113 (29.4) | 63 (32.0) | 50 (26.6) |  |
| Stage 2 | 73 (18.9) | 33 (16.7) | 40 (21.2) |  |
|  |  |  |  |  |
| Stage 1 & 2 | 186 (48.3) | 96 (48.7) | 90 (47.8) | 0.436 |

**Table B. Gender distribution of lifestyle variables**

| **Variable** | **Total** | **Men** | **Women** | ***p*-value** |
| --- | --- | --- | --- | --- |
| **Physical activity** |  |  |  | <0.0001* |
| No | 223 (57.9) | 93 (47.2) | 130 (69.1) |  |
| Yes | 162 (42.1) | 104 (52.8) | 58 (30.9) |  |
| **Smoking** |  |  |  | 0.613 |
| Never | 195 (50.6) | 95 (48.2) | 100 (53.2) |  |
| Formal | 120 (31.2) | 65 (33.0) | 55 (29.3) |  |
| Current | 70 (18.2) | 37 (18.8) | 33 (17.5) |  |
| **Alcohol use** |  |  |  | 0.935 |
| No | 144 (37.4) | 74 (37.6) | 70 (37.2) |  |
| Moderate | 171 (44.4) | 86 (43.7) | 85 (45.2) |  |
| Excessive | 70 (18.2) | 37 (18.7) | 33 (17.6) |  |
| **Consumption of sweet drinks** |  |  |  | 0.554 |
| No | 161 (41.8) | 83 (42.1) | 78 (41.5) |  |
| Moderate | 116 (30.1) | 55 (27.9) | 61 (32.4) |  |
| Excessive | 108 (28.1) | 59 (30.0) | 49 (26.1) |  |
| **Consumption of salty processed foods** |  |  |  | 0.414 |
| No | 175 (45.5) | 96 (48.7) | 79 (42.0) |  |
| Moderate | 130 (33.8) | 63 (32.0) | 67 (35.6) |  |
| Regular | 80 (20.7) | 38 (19.30 | 42 (22.4) |  |
| **Salt use** |  |  |  | 0.165 |
| None | 160 (41.6) | 88 (44.7) | 72 (38.2) |  |
| Moderate | 115 (29.9) | 61 (31.0) | 54 (28.7) |  |
| Excessive | 110 (28.6) | 48 (24.4) | 62 (33.1) |  |
| **Consumption of fruits** |  |  |  | 0.909 |
| No | 126 (32.7) | 65 (33.0) | 61 (32.4) |  |
| Yes | 259 (67.3) | 132 (67.0) | 132 (67.6) |  |
| **Consumption of vegetable** |  |  |  | 0.358 |
| No | 89 (23.5) | 42 (21.5) | 47 (25.5) |  |
| Yes | 290 (76.5) | 153 (78.5) | 137 (74.5) |  |

*Chi square test significant at *p*-value <0.05

**Table C. Gender distribution of anthropometric measurements**

| **Variable** | **Total** | **Men** | **Women** | ***p*-value** |
| --- | --- | --- | --- | --- |
| Body mass index |  |  |  | <0.0001* |
| Underweight | 21 (5.5) | 19 (9.6) | 2 (1.1) |  |
| Normal | 186 (48.3) | 126 (64.0) | 60 (31.9) |  |
| Overweight | 102 (26.5) | 39 (19.8) | 63 (33.5) |  |
| Obese | 76 (19.7) | 13 (6.6) | 63 (33.5) |  |
| Waist circumference | |  |  | <0.0001* |
| Normal | 220 (57.1) | 172 (87.3) | 48 (25.5) |  |
| ≥94 cm (men)/≥80cm (women) | 165 (42.9) | 25 (12.7) | 140 (74.5) |  |
| Waist-to-hip ratio |  |  |  | <0.0001* |
| Normal | 240 (62.3) | 179 (90.9) | 61 (32.4) |  |
| ≥0.95 (men)/≥0.80cm (women) | 145 (37.7) | 18 (9.1) | 127 (67.6) |  |

*Chi square test significant at *p*-value <0.05
